# Deep Neural Network with Joint Distribution Matching for Cross-Subject Motor Imagery Brain-Computer Interfaces

**DOI:** 10.1155/2020/7285057

**Published:** 2020-02-23

**Authors:** Xianghong Zhao, Jieyu Zhao, Cong Liu, Weiming Cai

**Affiliations:** ^1^Faculty of Electrical Engineering and Computer Science, Ningbo University, Ningbo 315100, China; ^2^School of Information Science and Engineering, Zhejiang University Ningbo Institute of Technology, Ningbo 315100, China

## Abstract

Motor imagery brain-computer interfaces (BCIs) have demonstrated great potential and attract world-spread attentions. Due to the nonstationary character of the motor imagery signals, costly and boring calibration sessions must be proceeded before use. This prevents them from going into our realistic life. In this paper, the source subject's data are explored to perform calibration for target subjects. Model trained on source subjects is transferred to work for target subjects, in which the critical problem to handle is the distribution shift. It is found that the performance of classification would be bad when only the marginal distributions of source and target are made closer, since the discriminative directions of the source and target domains may still be much different. In order to solve the problem, our idea comes that joint distribution adaptation is indispensable. It makes the classifier trained in the source domain perform well in the target domain. Specifically, a measure for joint distribution discrepancy (JDD) between the source and target is proposed. Experiments demonstrate that it can align source and target data according to the class they belong to. It has a direct relationship with classification accuracy and works well for transferring. Secondly, a deep neural network with joint distribution matching for zero-training motor imagery BCI is proposed. It explores both marginal and joint distribution adaptation to alleviate distribution discrepancy across subjects and obtain effective and generalized features in an aligned common space. Visualizations of intermediate layers illustrate how and why the network works well. Experiments on the two datasets prove the effectiveness and strength compared to outstanding counterparts.

## 1. Introduction

Brain-computer interfaces (BCIs), which set up a direct way from thought to realization, have provided us an imaginative future and have been paid great attention to [1–4]. As one important role of BCI families, motor imagery BCIs have witnessed great developments. For the reasons that there is no need of stimulations and the process is consistent with people's natural thinking habits, there have been many emerging applications, such as movement of a cursor or robotic limb and controlling of a wheelchair. However, motor imagery signals inherit the problem of nonstationary character of EEG (electroencephalogram) [5, 6]. Consequently, costly and boring calibration sessions must be proceeded before every test session for the same person [7–11]. It has long been known that a classifier with high accuracy for a subject could perform terribly for the same subject at a different time, which is called intersession or cross-session problem. Furthermore, the intersubject or cross-subject problem is more critical. Data from different subjects may have great discrepancy between each other, and the statistical distribution varies across subjects much more than that across sessions [10, 11]. This makes the cross-subject problem more difficult to handle. Other persons' data usually are discarded because only a few algorithms can take advantage of them. It is really a waste of time and resources.

One initial approach to get over this problem was to fix the classification rule beforehand and trained the patients to force brain activity to conform to this rule. For instance, subjects were trained to modulate and control the bandpower of their EEG signal [12, 13]. These methods pose great pressure on BCI users and take much time for users to fulfil the requirements.

To overcome this limitation, several groups introduced machine learning, especially transfer learning methods for adapting BCIs to target subjects [7–11, 14–18]. In recent years, several groups have started explicitly modelling such variations to exploit the common structure that is shared between multiple subjects. Several works explored data from other subjects (called source subjects, the data from them are called source data), in order to regularize common spatial patterns (CSP), ultimately to make the estimation of covariance matrix more unbiased and filters more effective for target subjects (the data of them are called target data) [14–16]. Other works constructed filter bank to extract more abundant features, selected them according to some designed rules, and then ensembled them to obtain high performance [8, 17, 18]. There are also researchers transforming features from different subjects into another space and making them more similar [9–11, 18]. They successfully managed to learn more common decoding rules with high accuracy utilizing both source and target data.

Most methods above learn a new shallow representation model by which the domain discrepancy can be explicitly minimized. However, without learning deep features to suppress domain-specific exploratory factors of variations, the transferability of shallow features is restricted by task-specific structures [19, 20]. Deep learning has been proved not only to have more power to extract compact and deep-level features but also possess more strength to represent the task. It has won great achievements in many fields, especially including EEG decoding [21–34]. For instance, we focus anomaly detection [21], visual evoked potentials [22], P300 detection [24], workload analysis [25], error-related negativity responses (ERN) [30], movement-related cortical potentials (MRCP) [30], attentional information [35], and motor imagery tasks [26–34]. Deep neural networks are paid more and more attention for motor imagery tasks. These methods explored deep neural networks to obtain more compact and effective features. However, they need much more data for training. Usually, data from different subjects are pooled together and fed directly into the network, regardless of the statistical distribution discrepancy across subjects. It will result in obvious deterioration of the network [7, 34, 36].

**Figure 1:**
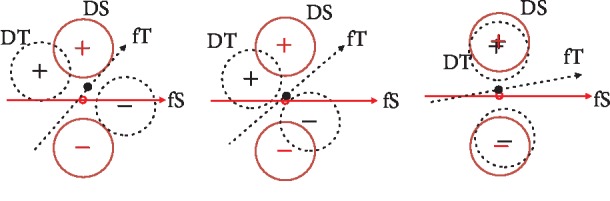
Illustration for joint distribution adaptation. DS: source domain, red solid circle; DT: target domain, black dashed circle. +: centroid of positive class; -: centroid of negative class. Red hollow circle: centroid of source data; black solid circle: centroid of target data. fS: discriminative line for source data; fT: discriminative line for target data. Marginal domain adaptation (MDA) utilizing MMD makes the centroid of source data (red hollow circle) and that of the target data (solid black circle) closer. Joint distribution adaptation aligns source and target data according to the class they belong to. It makes the discriminative lines of source and target most similar, and the classifier trained with source data will be transferred to target most effectively.

In this work, we will not only explore deep learning methods to learn more compact and deep-level features but also utilize domain adaptation methods to alleviate the discrepancy across data from source and target. This theory will help make full use of other persons' historical data and cut off the training efforts for target users as much as possible. It will benefit BCI users the most and make BCI plug-and-play in realistic application scenarios. The main contributions of this paper are as follows. Firstly, a new joint distribution distance measure, called joint distribution discrepancy (JDD), is proposed. It can effectively measure the joint distribution discrepancy between data from different subjects. It can be added to the deep neural network as an effective regularized part. We also propose the idea that the crucial domain adapting method is to adapt both the marginal distribution and joint distribution between different domains. It can be illustrated as Figure 1. From the figure, it is found that the performance of classification would be bad when only the marginal distributions of the source and target are made closer, since the discriminative directions of the source and target domains may still be much different. Our idea is that it is indispensable to minimize the discrepancy between the joint distributions of source and target, in order to make the classifier trained in the source domain perform best in the target domain. JDD can make source and target data aligned and close according to the class they belong to ultimately make the discriminative line of source data close and similar to that of target data. The model trained on source will be transferred to the target better. On the contrary, marginal domain adaptation (MDA) alone makes the marginal distribution closer, may not lead to good classification results, and is not as effective as our idea. Without JDD alignment, the data from the source and target cannot be merged together. The discriminative directions between source and target data will be much different and classifier trained with source data will not work well for target data. It is also proved that JDD have a reverse relationship with classification in total. When JDD decreases, the classification accuracy will grow up. Secondly, a deep neural network with joint distribution matching for cross-subject motor imagery BCI is proposed. It explores both marginal and joint distribution adaptations to fine-tune the network, in order to alleviate all these discrepancies across subjects and obtain effective features in an aligned common space. Visualizations of intermediate layers illustrate how and why the network works well. Model trained with source data is transferred directly to the target subjects. Experiments on the two datasets prove the effectiveness and strength compared to counterparts.

## 2. Related Works

Transfer learning methods are designed to make the training data domain closer to the test domain and have got great achievements in many areas, such as image, audio, and text processing [19, 20, 37–41]. Due to the time-varying and nonstationary characters of EEG signals, the training data are statistically different from the test data, and the performance of the classifier obtained from the training data will degrade significantly, especially when test data come from different subjects [7–11, 14–18]. Considering these, transfer learning methods are adopted to make improvements for BCI. Song and Yoon exploited train and test data together to make the estimation of variance matrix more accurate for test data [14]. Lotte and Guan introduced a unifying theoretical framework to design four regularized methods and alleviate the bias of estimation of variance matrix [15]. These methods explore both source and target data to extract more stationary CSP (common spatial patterns) features and make progress in classifying tasks. Park and Lee obtained a robust and adaptive filter bank from source subjects and then learned the classifiers corresponding to these filters banks and then employed a two-level ensemble strategy to reach a single decision output [16]. Park et al. in [8] firstly divided EEG data with a filter bank. Secondly, the regularized CSP (R-CSP) is applied to them. Features were selected according to mutual information. Finally, an ensemble classifier was trained to obtain results. Zanini et al. proposed a transfer learning method based on the Riemannian geometry framework [10]. It utilized affine transform to the covariance matrix of sessions or subjects. Like a clustering process, it obtained a covariance matrix as the center and made data from different sessions/subjects more similar. Then the classification was performed based on a mixture of Riemannian Gaussian distributions defined on the manifold. It transformed data from different sessions and subjects to a new common space and achieved outstanding results. Rodrigues et al. in [11] proposed a method called RPA. It was based on Procrustes analysis for matching the statistical distributions of two datasets. Symmetric positive definite matrices (SPD) as statistical features and geometrical operations on the data points were utilized. Improvements in transfer learning via RPA by performing classification tasks on simulated data and on eight publicly available BCI datasets were assessed.

Domain adaptation theory can also play an important role in subject transfer problems. It can alleviate the differences between subjects. Pan et al. in [42] presented a method which not only reduced the distance between the source domain and target domain using maximum mean discrepancy (MMD) [43] but also tried best to preserve the variance of the original data. Tao et al. in [37] first constructed a generalized measure for domain adaptation on reproducing kernel Hilbert spaces (RKHS) by simultaneously considering both the projected marginal discrepancy and the projected maximum distribution scatter discrepancy between the source and the target domain. Long et al. in [38] decomposed the joint distribution as *P*(*x*, *y*) = *P*(*x* | *y*)*p*(*y*), and then both the differences of *P*(*y*) and *P*(*x* | *y*) for source and target domain were simultaneously decreased in order to match the joint distribution *P*(*x*, *y*). Firstly, an initial classifier provided pseudolabels to the target data using MMD. Then, the difference of the conditional distribution *P*(*x* | *y*) between source and target was minimized to improve the previous classifier; process was iterated until convergence. The algorithm performed well on many text and image datasets. It achieved very good results in comparison and the algorithm was called ARRLS. The idea is similar to ours. The difference between ours and ARRLS is that ours utilizes the proposed JDD to reduce the joint distribution discrepancy in RKHS straightforwardly.

Previous methods exploit shallow networks to match the domains of a single level; deep neural networks are good at extracting multilevel and compact features and will have better descriptions for specific tasks [26–34]. Many deep learning approaches are applied to decode EEG signals. Tabar and Halici exploited CNN and SAE (stacked autoencoders) to classify motor imagery EEG signals [26]. It combined time, frequency, and space information of motor imagery data into deep models and achieves outstanding results in BCI competition IV dataset 2b. Schirrmeister et al. in [30] first investigated different deep architectures and then introduced a compact fully convolutional network called EEGNet for four different tasks. Compared with the corresponding works, they performed averagely the best over different datasets. They claimed that they suggested a common simplified architecture. It can provide robust performance across many different BCI modalities. It is very effective in our experiments and is chosen as one of the baselines. In a whole, these methods exploit mainly CNN and its corresponding structures. Other types of deep neural networks extend their potential on motor imagery signals. Wang et al. in [27] proposed a deep framework based on long short-term memory (LSTM) networks. One-dimensional-aggregate approximation (1D-AX) was employed to extract an effective signal representation for LSTM networks. Meanwhile, the channel weighting technique was further deployed to enhance the effectiveness inspired by CSP. Lu et al. in [29] proposed a novel deep learning scheme based on restricted Boltzmann machine (RBM). Specifically, frequency domain representations obtained via fast Fourier transform (FFT) and wavelet package decomposition (WPD) were obtained to train the three RBMs. These RBMs were then stacked up with an extra output layer to form the frequential deep belief network (FDBN). The output layer employed the softmax regression to accomplish the classification task.

Recent studies reveal that a deep neural network with domain adaptation technique can learn both deeper and more transferable features. It can generalize well to the novel domain [19, 20]. Tzeng et al. in [39] proposed a DDC model that adds an adaptation layer and a dataset shift loss to the deep CNN for learning a domain-invariant representation. While the performance was improved, DDC only adapts a single layer of the network and Long et al. furthered this idea [20]. Multilevel features are matched utilizing multiple-kernel MMD. Long et al. also exploited a better way to reduce the computation cost for MMD and obtained a better result. Yosinski et al. in [40] revealed that feature transferability got worse on stack-behind layers and significantly drops on the last layers; hence, it was critical to adapt multiple layers instead of only one layer. Jian et al. in [41] proposed an adversarial representation learning approach to learn high-level representations that are both domain-invariant and target-discriminative, in order to tackle the cross-domain classification problem. It was inspired by Wasserstein generative adversarial networks and obtained good results in 4 common domain adaptation datasets.

The discussed methods above focus on images, text, and so on. There are also a few works to cover deep domain networks for BCI. Fahimi et al. in [35] developed an end-to-end deep CNN to decode the attentional information from EEG time series. They also explored the consequence of input representations on the performance of deep CNN by feeding three different EEG representations into the network. Additionally, intersubject transfer learning techniques were performed as a classification strategy. It is called CNN-subject adaptation and is called CNN-SA in short in our paper. Farshchian et al. in [36] implemented various domain adaptation methods to stabilize the interface over significantly long time, including canonical correlation analysis, minimizing the Kullback-Leibler divergence of the empirical probability distributions. These two methods provided a significant and comparable improvement in the performance of the interface. However, the implementation of an adversarial domain adaptation network trained to match the empirical probability distribution outperformed the two methods based on latent variables, while requiring remarkably fewer data. Tan et al. in [44] modeled cognitive events by characterizing the data using EEG optical flow, which is designed to preserve multimodal EEG information in a uniform representation. After that, a deep transfer learning framework, which was suitable for transferring knowledge by joint training, was constructed. It contained an adversarial network and a special loss was designed.

## 3. Methods

Previous methods applied for EEG decoding either utilize deep networks alone or exploit shallow domain adaptation networks to explicitly minimize the domain discrepancy. It can be imagined that the performance may be enhanced, if deep neural networks can be combined with the transfer learning methods above. By learning deep and high compact features with deep networks and domain adaptation, domain-specific exploratory factors of variation will be suppressed and generalized; the transferability of deep features will not be restricted by subject-specific structures [20, 39]. Therefore, a deep neural network with domain adaptation is proposed. Meanwhile, as previous works shown [8, 14–17, 45], the most important characters of motor imagery signals are ERD (event-related synchronization) and ERS (event-related desynchronization). CSP is considered as the most effective and popular method. However, in the conventional classification process, the discriminating operation is separated from feature extracting operations. The features extracted cannot assure the best performance of classification. If CSP is adopted in deep networks along with the discriminative process, it is possible to grantee the best classification results. CSP is aimed at finding spatial filters which maximize (or minimize) the variance of the projected data points of one class while the other is minimized (or maximize). Given motor imagery signal matrices *X*_1_ and *X*_2_ (channels by samples, ch × *T* in short), which belong to class 1 and class 2, respectively, the target function of the optimization can be described as formula (1) mathematically. The mean of *X*_*i*_ was removed before fed to the following equation:

(1) $$\begin{array}{c} \underset{w}{\arg\min}\;J_{1}\left(w\right)=\frac{w^{T}X_{1}X_{1}^{T}w}{w^{T}X_{2}X_{2}^{T}w}. \end{array}$$

**Figure 2:**
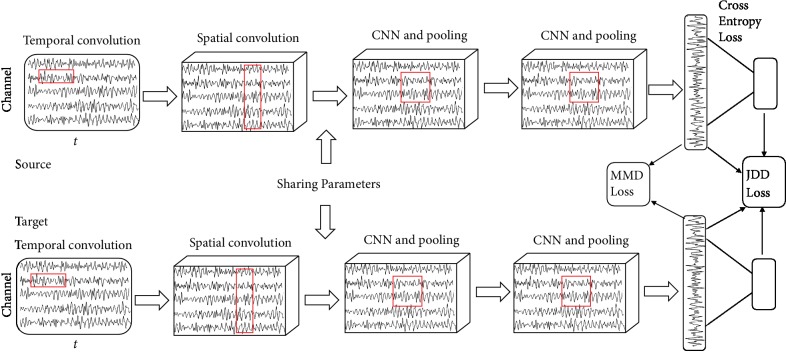
Proposed deep network structure for domain adaption (DCJNN).

The vectors obtained from above are called CSP filters, which can extract energy features and make the differences of the two classes maximized. Operations for *w*^*T*^*X*_1_ is much alike one-dimensional convolution in deep learning. The number of filters we pursue is the number of kernels for convolution. Considering this, a deep CSP neural network with joint distribution adaptation (DCJNN) is proposed. The detailed architecture and settings are as Table 1 and Figure 2. Rectified linear unit (ReLU) function is selected as activation function, which is defined as ReLu(*x*) = ln(1 + *e*^*x*^).

The first layer utilizes one-dimensional convolutional kernel to realize time-domain filters for each channel. What the filters want to accomplish is to filter the EEG time series and divide them into different bands according to the classification task, such as mu rhythm and beta rhythm, which are the important EEG bands for motor imagery task. Frequency bands should be carefully chosen, the reason is that the performance of the CSP algorithm depends much on the frequency bands [8, 18, 45]. Proper frequency bands and time-domain features are expected to be caught automatically in this layer. This layer also includes a batch normalization (BN) block and a dropout block. They can help accelerate the training process and improve the robustness of the network, which is similar as each of the following layers. Dropout block in fact increases the diversity of input samples and prevents the model from overfitting.

The second layer is aimed at pursuing spatial filtering like the CSP algorithm in formula (1) and extracting deep-level features combining time, spatial, bandpower, and intersubject characteristics. Similarly, as the first layer, the second layer employs one-dimensional convolutional kernel. The differences between them are that the second layer focuses on filtering EEG signals in the spatial domain. It is worth noting that it is specifically designed to produce spatial filters and enhance the signal to signal-plus-noise ratio of the EEG signal of interest. The third and fourth layers exploit 2D convolution to pursue both the spatial and frequency domain features. The last two layers are the same as conventional CNN networks. The features are flattened, and a full-connection layer is constructed. The outputs are the vectors for classification. The dimension *N* is the number of classes.

At the fifth layer, maximum mean discrepancy (MMD) is exploited to adapt the marginal distribution between the source and target. They try their best to make the marginal distribution of source features aligned to that of target. The empirical MMD can be calculated as follows:

(2) $$\begin{array}{c} \overset{\sim }{\text{MMD}}{\left(P_{s},P_{t}\right)}^{2}={\left(\frac{1}{\text{ns}}\overset{\text{ns}}{\underset{i=1}{\sum }}\phi \left(x_{i}^{s}\right)-\frac{1}{\text{nt}}\overset{\text{nt}}{\underset{j=1}{\sum }}\phi \left(x_{j}^{t}\right)\right)}^{2}=\text{trace}\left(K_{x}\circ W_{1}\right). \end{array}$$

*P*_*s*_ and *P*_*t*_ represent marginal distribution of source and target, respectively. *x*_*i*_^*s*^ and *x*_*j*_^*t*^ denote the features at the fifth layer for the *i*th and *j*th sample of source and target data, respectively. *K*_*x*_ stands for gram matrix of data including source and target. “o” denotes the Hadamard product. In formula (2), *W*_1_ = [[(1/ns) ⋯ (1/ns)]_1×ns_, [−(1/nt) ⋯ −1/nt]_1×nt_]^T^ · [[(1/ns) ⋯ (1/ns)]_1×ns_, [−(1/nt) ⋯ −1/nt]_1×nt_]. The parameters ns and nt stands for the number of source samples and target samples. They equal to ba. ba stands for the number of batch data fed into the network each time. The detailed operation for MMD loss is as follows. Firstly, a batch of source samples are fed to the neural network. They flow through each layer and obtain results. *x*_*i*_^*s*^ represents the outputs of the fifth layer for *i*th source sample. After that, the networks are frozen and a batch of samples from the target similarly flow through all the layers. *x*_*j*_^*t*^ represents the outputs of the fifth layer for *j*th target sample. At last, the MMD loss is computed as formula (2) and constitute the total loss as formula (5). The corresponding gradients are back propagated to improve the network parameters.

**Figure 3:**
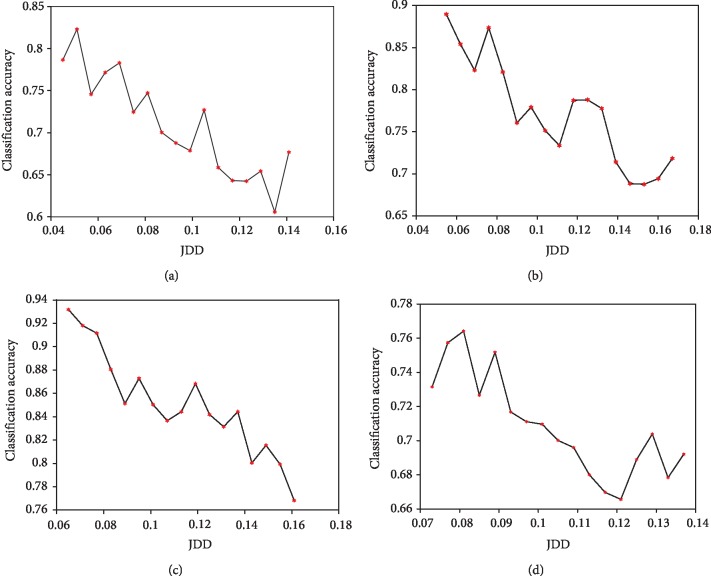
Relationship between classification accuracy and JDD. The horizontal and vertical ordinate represents the JDD and classification accuracy, respectively. (a) GrazA subj1. (b)Graz subj3. (c) Giga subj2. (d)Giga subj3.

Similarly, the following JDD loss can be calculated. JDD block explores the predicted results of source and target along with the features, in order to make the joint distribution between source and target aligned to each other. If we want to compute the differences of joint distribution *P*(*x*, *y*) between source and target domain, our idea is to find a way to measure the discrepancy of two joint distributions. Inspired by maximum mean discrepancy and kernel embedding theory, JDD is defined as Definition 1. JDD takes both features and labels into consideration and measures the joint distribution discrepancy between the source and target. It is a more accurate and effective measure when we explore source labelled data to predict target data.  Figure 3 illustrates the JDDs have a reverse relationship with the classification accuracies. It is a very effective distance measure to align the source and target domain.

**Definition 1 .**

Joint distribution discrepancy (JDD)

(3) $$\begin{array}{c} \text{JDD}\left({ℱ}_{1},{ℱ}_{2},P,Q\right)=\underset{\left\|f\right\|\leq 1,f\in {ℱ}_{1};\left\|g\right\|\leq 1,g\in {ℱ}_{2}}{\sup}\left[\int f\left(x\right)g\left(y\right)dP\left(x,y\right)-\int f\left(x\right)g\left(y\right)dQ\left(x,y\right)\right]. \end{array}$$

In which *x*, *y*, and *P*(*x*, *y*) represent samples, their corresponding predicted labels, and joint distribution, the same is to *Q*(*x*, *y*). It can be deduced that the discrepancy JDD(*ℱ*_1_, *ℱ*_2_, *P*, *Q*) equals zero if and only if joint distributions *P*(*x*, *y*) and *Q*(*x*, *y*) are equal to each other. The smaller JDD indicates the two joint distributions lie closer to each other. Therefore, JDD(*ℱ*_1_, *ℱ*_2_, *P*, *Q*) can be exploited as an efficient way to measure the discrepancy between two joint distributions *P*(*x*, *y*) and *Q*(*x*, *y*). In our algorithm, *P*(*x*, *y*) and *Q*(*x*, *y*) represent joint distribution for source and target data, respectively. Therefore, JDD measures the joint distribution discrepancy between the source and target.

Empirical unbiased estimation of JDD can be calculated as follows:

(4) $$\begin{array}{c} \overset{\sim }{\text{JDD}}{\left({ℱ}_{1},{ℱ}_{2},P_{s},P_{t}\right)}^{2}={\left\|\frac{1}{\text{ns}}\overset{\text{ns}}{\underset{i=1}{\sum }}\phi \left(x_{i}^{s}\right)⊗\varphi \left(y_{i}^{s}\right)-\frac{1}{\text{nt}}\overset{\text{nt}}{\underset{i=1}{\sum }}\phi \left(x_{i}^{t}\right)⊗\varphi \left(y_{i}^{t}\right)\right\|}_{\text{HS}}^{2}=\frac{1}{{\text{ns}}^{2}}\overset{\text{ns}}{\underset{i=1}{\sum }}\overset{\text{ns}}{\underset{j=1}{\sum }}K_{1}\left(x_{i}^{s},x_{j}^{s}\right)K_{2}\left(y_{i}^{s},y_{j}^{s}\right)+\frac{1}{{\text{nt}}^{2}}\overset{\text{nt}}{\underset{i=1}{\sum }}\overset{\text{nt}}{\underset{j=1}{\sum }}K_{1}\left(x_{i}^{t},x_{j}^{t}\right)K_{2}\left(y_{i}^{t},y_{j}^{t}\right)-\frac{2}{\text{ns}\cdot \text{nt}}\overset{\text{ns}}{\underset{i=1}{\sum }}\overset{\text{nt}}{\underset{j=1}{\sum }}K_{1}\left(x_{i}^{s},x_{j}^{t}\right)K_{2}\left(y_{i}^{s},y_{j}^{t}\right)=1^{T}\left(K_{x}\circ K_{y}\circ W_{1}\right)1, \end{array}$$

where “o” stands for Hadamard product; *K*_*x*_ and *K*_*y*_ stand for gram matrix of data and predicted labels including source and target domains, respectively. *y*_*i*_^*s*^ and *y*_*j*_^*t*^ represent the labels predicted by the networks, for *i*th source samples and *j*th target samples, respectively. Therefore, it is found the joint distribution discrepancy between source and target can be calculated only by the corresponding kernel gram matrix. It is convenient and effective. In our paper, all the kernels we adopt are the RBF kernels.

To summarize, our objective loss of the neural network can be as formula (5). The first part of the loss denotes the supervised loss of source data. In this paper, cross-entropy loss is applied. *γ*_*J*_ and *γ*_*M*_ denote the trade-off parameters. The second and third parts are JDD as formula (4) and MMD loss as formula (2).

(5) $$\begin{array}{c} \text{loss}=\int_{X\times Y}V\left(y,f\left(x\right)\right){dP}_{s}\left(x,y\right)+\gamma_{J}\overset{\sim }{\text{JDD}}{\left({ℱ}_{1},{ℱ}_{2},P_{s},P_{t}\right)}^{2}+\gamma_{M}\overset{\sim }{\text{MMD}}{\left(P_{s},P_{t}\right)}^{2}. \end{array}$$

## 4. Experiments and Results

The first EEG dataset used in the BCI Competition 2008 and called Graz dataset A (GrazA in short) is provided by the Graz Institute [46] (URL: http://www.bbci.de/competition/iv/). This dataset consists of EEG data from 9 subjects (called “subj1” to “subj9”). The cue-based BCI paradigm consisted of four different motor imagery tasks, namely, the imagination of movement of the left hand (class 1), right hand (class 2), both feet (class 3), and tongue (class 4). Two sessions on different days were recorded for each subject. Each session is comprised of 6 runs separated by short breaks. One run consists of 48 trials (12 for each of the four possible classes), yielding a total of 288 trials per session. The signals were sampled with 250 Hz and bandpass filtered between 0.5 Hz and 100 Hz. The sensitivity of the amplifier is set to 100 *μ*V. An additional 50 Hz notch filter was enabled to suppress the line noise. In our paper, for example, when we want to study “subj1,” then “subj1” and its data are called target subject and target data, respectively. The other eight subjects and their data are called source subjects and source data. The settings are similar to the second dataset in our paper. As preprocessing, each channel of the EEG data was bandpass filtered causally to 4 Hz~40 Hz by a Chebyshev type 2 filter of order five (stop-band attenuation of 20 dB), and then an epoch of 0.5 s to 5 s relative to the stimulus is used in our paper. Therefore, we have a dataset of 9 by 288 by 64 by 450 (subjects by trials by channels by time points). EEG signals are typical nonstationary and time-varying data.  Figure 4 in [10] demonstrates that the data of all subjects are depicted together. It indicates that there are great discrepancies among data from different subjects. They vary a lot across subjects and show very bad separation among subjects.

The second dataset adopted in this paper was supplied by Handong Global University [47], called GigaDataset (http://gigadb.org/dataset/100295) in our paper. 52 healthy subjects (26 males, 26 females; mean age: 24.8 ± 3.86 years, called subj1~subj52) participated. The subjects were asked to imagine left hand or right hand movement. At the beginning of each trial, a cross appeared for 2 s, and then text indicating left or right was shown for 3 s. Subjects were asked to imagine left or right hand movement according to the presented direction at the motor imagery phase. Right after the motor imagery phase, a cross appeared for 2 s again. Thus, the total time of each trial was 7 s and the intertrial interval was set randomly to between 0.1 and 0.8 s. 68 electrodes (in which 64 were for EEG) were utilized to record the motor imagery signals. The sampling rate was 512 Hz. In this paper, the data of randomly chosen six subjects, namely, subj2, subj9, subj11, sub13, subj21, and subj36, were explored and all the 64 EEG channels were used. As preprocessing, epoch of 0.4 s to 3 s after the cue was utilized and was bandpass filtered to 4 Hz~30 Hz. After that, they are downsampled to 100 Hz. There were 200 trials for each subject, one half was for imagining the left and the other half was for imagining the right. Therefore, we have a dataset of 6 by 200 by 64 by 260 (subjects by trials by channels by time points).  Figure 5 demonstrates the brain electrical activity mapping (BEAM) for imagining the right hand for subjects 2 and 9. the BEAMs actually indicate the energy distribution or the activity of neurons across the brain. The same row belongs to the same subject performing the same task at different times and the different rows belong to different subjects, respectively. It is clear that not only different subjects show very great differences when performing the same task but also the same subjects make differences performing the same task at different times. Distribution of EEG data is very much time-varying and different from subject to subject. It is in great need of distribution matching.

As illustrated in the third section, the second layer of our network tries to play a similar role as common spatial filters. After training the networks utilizing GigaDataset, the absolute values of some filters are demonstrated as Figure 6. The value of spatial filters represents the importance of the corresponding channel or the position of the EEG. The greater the value is, the more important the corresponding channel is. From the figure, totally speaking, it can be found out that weight of filters corresponding to the right part is stronger than the left for the upper three. The other three are on the contrary. It tells us the network learns to extract spatial filters as CSP filters. Meanwhile, it verifies the theory of ERD/ERS for motor imagery signals [8, 16, 45]. The upper three show the right part of the head have stronger reflections, and the down three show the contrary. It can be deduced that the upper three can be taken as the CSP filters for imagining the left hand and the down three can be taken as the CSP filters for imagining right hand [45]. It indicates that our proposed network can learn to obtain CSP-like filters and to extract CSP features with iterations, as expected. It will be effective at the classification stage.

In order to demonstrate the power of our algorithm JDD further, the data of subj1 and subj2 in GrazA are visualized as Figures 7(a) and 7(b), respectively. Red circles and hexagrams represent class 1 and class 2 data of subj1, respectively. Black circles and hexagrams represent class 1 and class 2 data of subj2, respectively. Red and black lines represent the discriminative lines for subj1 and subj2, respectively. In Figure 7(a), firstly, data from subj1 and subj2 are independently feed into our proposed network in which the JDD constraints are removed. Outputs from the fifth layer (flatten layer) are taken as initial features. After that, kernel principal component analysis (KPCA) is adopted and the dimension is reduced to 2 [47, 48]. The discriminative lines are obtained by linear discriminative analysis on the two-dimensional data. Figure 7(a) illustrates the features extracted without JDD alignment. It is found that data from different subjects have great discrepancy between them. The discriminative line of subj1 is very different from that of subj2. It can be deduced that the classification performance will be not good when they are forced to being trained together. It will be also very bad that the classifier trained on source data is directly applied to the target data.

However, when the same processing method is adopted, excluding that JDD constraints are added to the neural network as proposed, the results are illustrated as Figure 7(b). It can be found that the discrepancy between data from subj1 and subj2 is much less and the data are merged together. Furthermore, the discriminative lines of the two subjects are much closer and more similar. Under this condition, the performance will be good when the classifier trained on source data is directly applied on the target data. It will do great good to the performance of classification, since the trained classifiers from different subjects will be well transferred. It indicates that our method works well.

Four outstanding algorithms are selected as baselines. ARRLS in [38] is very similar with ours since it takes both marginal and conditional distribution into account. It aims to match the joint distribution across subjects. It is not a deep learning method, so as AT-GM-b in [10]. AT-GM-b in [10] is also an outstanding algorithm. It transforms the data from different subjects into a common space, which aims to make data from subjects closer under the Riemannian framework. They are not in a deep learning manner, but still they are very representative and effective transfer learning methods for BCI. CNN-SA in [35] and EEGNet in [30] explore a deep learning framework to work out motor imagery tasks. EEGNet is designed to work out different kinds of data for BCI, including motor imagery BCI. Until now, it is a very effective and competitive method. Its network structure is similar to ours but ours possesses marginal and joint distribution adaptation units. Ours focuses on how to alleviate domain discrepancy across subjects and have more power in domain adaptation. CNN-SA develops an end-to-end deep CNN to decode the attentional information from EEG time series. They also explore the consequence of input representations on the performance of deep CNN by feeding three different EEG representations into the network. Additionally, intersubject transfer learning techniques are performed as a classification strategy. Details can be found in [35]. It is a deep learning method exploiting subject transfer technique, and so it is chosen as another baseline. In our paper, the data we try to classify are taken as target data and the data of other subjects are taken as source data, which are utilized to train a classifier. We run experiments on GrazA and GigaDataset independently and two networks are trained. Taking dataset GrazA as an example, when we want to classify the data of subj1, then the data from subj2 to subj9 are taken as source data and training dataset. The data of subj1 is the testing data or called target data. Parameters *γ*_*J*_ and *γ*_*M*_ in formula (5) are chosen in {0.01, 0.1, 0.2, 0.5, 1, 2, 5, 10, 20}. They are optimized through 5-fold cross validation. Training data are randomly divided into five parts, four parts are for training, and the remaining one is for validation. At each time, a batch of training samples (ba = 32) are randomly selected from the training dataset and fed to the neural network. The maximized epoch we run is set as 200. The same is for GigaDataset. The deep neural networks for CNN-SA and ours are constructed with TensorFlow 1.3.0 with GPU acceleration. The optimizer is chosen as the AdamOptimizer. Implements for EEGNet are used as provided in [49].

For GrazA, comparison results are illustrated as Figures 8(a)–8(c). The results vary across subjects. These methods are all competitive and effective. In a whole, our algorithm runs best for most conditions, six out of nine. The EEGNet wins the other three, including subj1, subj5, and subj6. For these three subjects, ours falls a little behind the champion, by 0.5%, 1.6%, and 7.1%, respectively, and still we outperform the other three baselines much. In a whole, mean and variance for classification results 69.6% and 15.1%, which indicates that our algorithm can be applied well across all the subjects, in an unsupervised domain adaptation manner. It averagely outperforms the counterparts by 8.2%, 7.3%, 8.5%, and 1.0%, respectively. The means of the other four algorithms' classification accuracies are 64.2%, 64.7%, 64.0%, and 68.9%, respectively. It is worth noting that GrazA is a four-category problem and the accuracy is not very low, although it can be further improved in the future. Furthermore, it is found that subjects can be divided into two categories. The accuracies of subj2, subj4, subj5, and subj6 are relatively low, about 50%, which are also found in [10]. The others perform very well, and the accuracies surpass 75%. Differences across subjects performing motor imagery are huge and obvious; the same thing happens for GigaDataset. It is also the reason why we want to apply domain adaptation for motor imagery. Under motor imagery tasks, it is not unusual that the performances of some subjects are relatively worse than others. One of the reasons is that they are not trained well for motor imagery. Another one is that some of them are not very good at this, however hard they try. It is usually called BCI blindness and it is another research topic worth studying.

For GigaDataset, the comparison results are illustrated as Figures 9(a) and 9(b). The results also vary across subjects. In a whole, our algorithm runs best for most conditions, four out of six. The EEGNet wins the other two, including subj9 and subj21. For these subjects, ours falls a little behind the champion, by 0.4% and 0.8%, respectively. And still we outperform the other three baselines greatly. EEGNet proves its strength and effectiveness. Considering the facts in GrazA, it can be deduced that domain adaptation may not work for all subjects due to the great diversity of data distribution. However, our algorithm outperforms the counterparts in a whole. Its strength and effectiveness across all the subjects have been proved. Its mean and variance for classification results 76.5% and 9.3%, which also indicates that our algorithm can be applied well across all the subjects, in an unsupervised domain adaptation manner. It averagely outperforms the counterparts by 5.8%, 7.1%, 4.4%, and 1.7%, respectively. The means of the other four algorithms' classification accuracies are 72.3%, 71.4%, 73.3%, and 75.2%, respectively.

Meanwhile, relationship between classification accuracy and JDD is also explored and the results are demonstrated as Figure 3. It can illustrate whether JDD works well and JDD's influence. The horizontal and vertical ordinate represents the JDD and classification accuracy, respectively. Figures 3(a)–3(d) represent the results for subj1 and subj3 in GrazA and subj2 and subj3 in GigaDataset, respectively. From the figure, it can be found the trends of JDD and classification accuracy are inverse with each other in a whole. That is to say, the classification accuracy will grow when JDD decreases. As in Figure 7, JDD plays an important role in aligning source and target data. It makes the source and target data merged together, and the discriminative lines of source and target data are closer and more similar to each other. Therefore, when JDD works, the performance of domain adaptation will grow better, which leads to better classification results.

## 5. Conclusions

**Figure 4:**
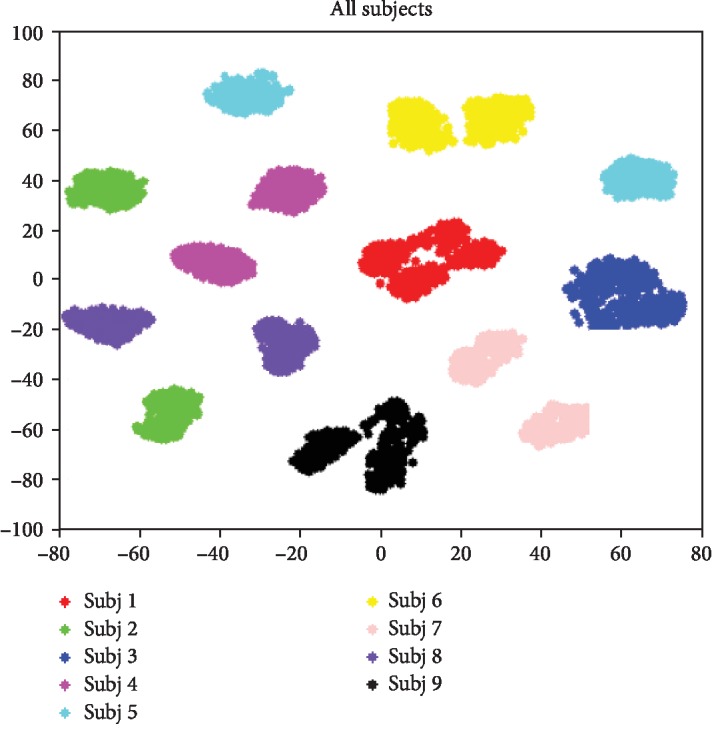
Motor imagery dataset: visualization of the original covariance matrices of all subjects [10].

**Figure 5:**
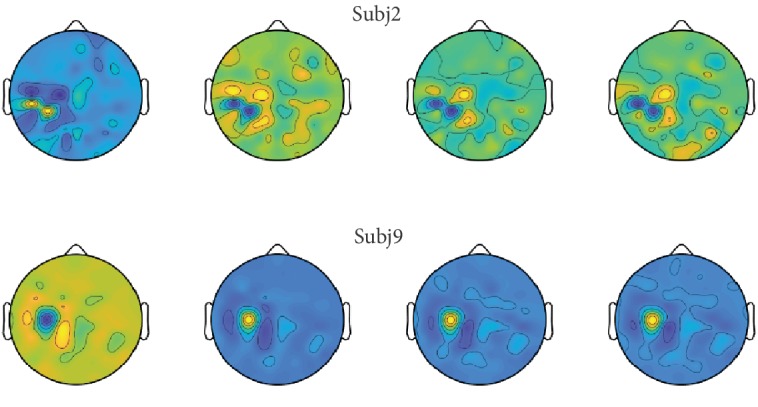
Brain electrical activity mapping when performing right hand imagery in GigaDataset.

**Figure 6:**
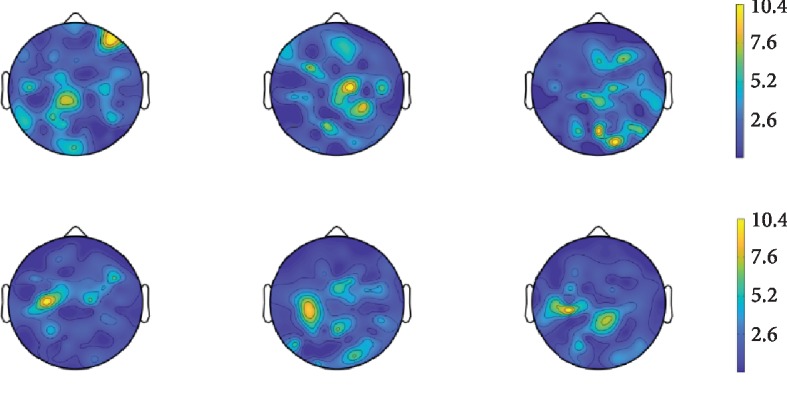
CSP filters obtained by the proposed neural network.

**Figure 7:**
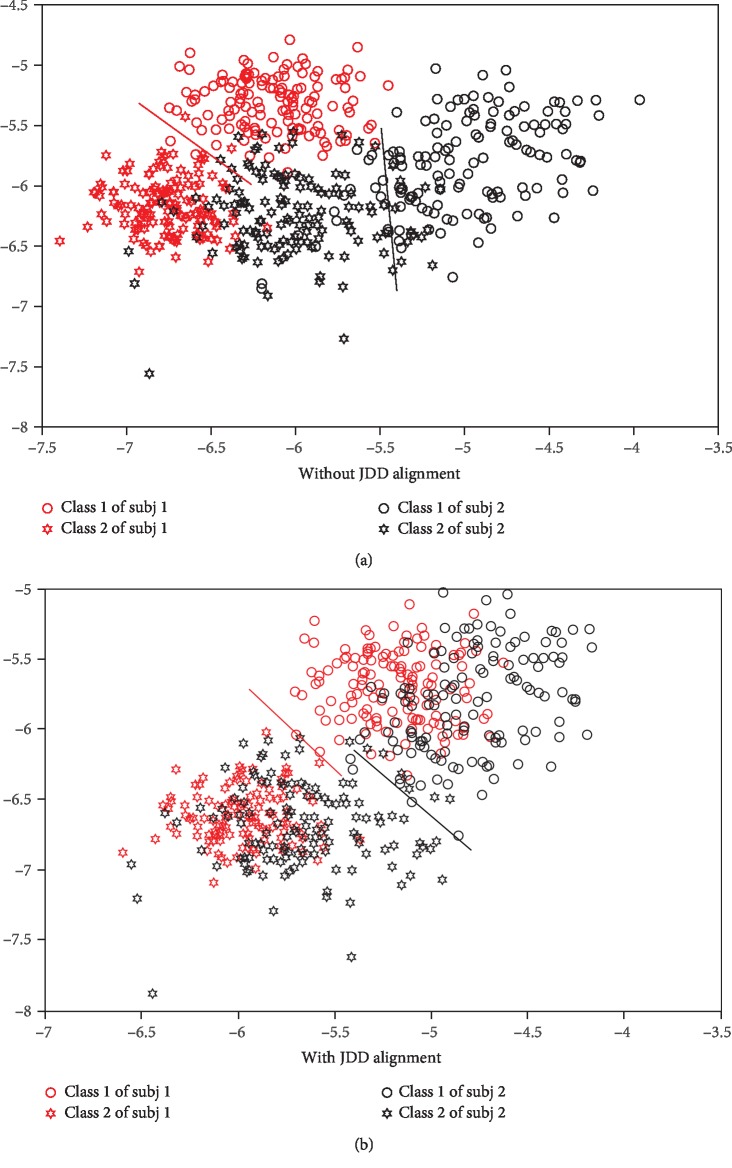
Visualization of motor imagery data. (a, b) Data after dimension reduction without and with JDD alignment, respectively. (a) Without JDD alignment, (b) With JDD alignment.

**Figure 8:**
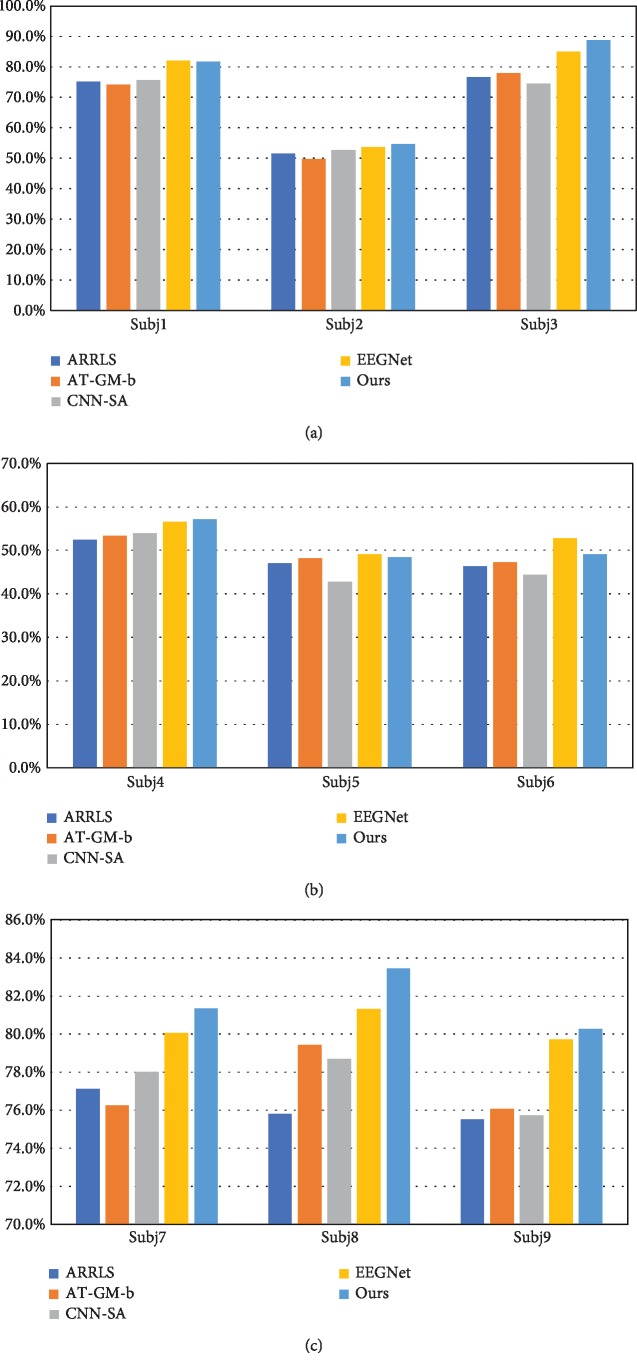
Comparison results for GrazA.

**Figure 9:**
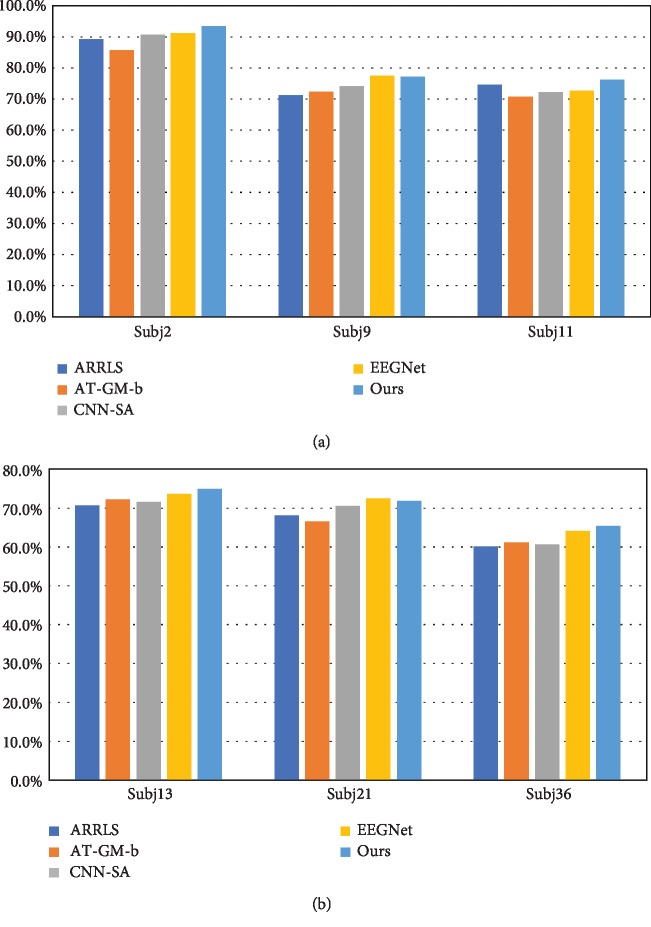
Comparison results for GigaDataset.

In this paper, we proposed a deep neural network with joint distribution matching for motor imagery brain-computer interfaces. Firstly, the nonstationary character of EEG, especially across different subjects, is analyzed and illustrated as Figures 4 and 5. Models trained with source subjects will not transfer as well as expected to target. Furthermore, it is found that performance of classification would be bad when only the marginal distributions of source and target are made closer, since the discriminative directions of the source and target domains may still be much different. It is indispensable that the joint distribution of the source and target should be aligned as Figure 1 illustrates. Therefore, a distance for measuring the joint distribution discrepancy (JDD) between source and target data is proposed. JDD takes both data and corresponding labels into consideration. Minimizing JDD between source and target can merge together and align data from different subjects, as Figure 7 proves. Moreover, JDD has an inverse relationship with classification accuracy in a whole. It is very useful for optimizing process. Secondly, a deep neural network with joint distribution matching is proposed. It explores both marginal and joint distribution adaptation to fine-tune the network, in order to alleviate all these discrepancies across subjects and obtain effective features in an aligned common space. Visualizations of intermediate layers illustrate how and why the network works well. Model trained with source data is transferred directly to the target subjects. Experiments on the two datasets prove the effectiveness and strength compared to outstanding counterparts. For grazA, it averagely outperforms the counterparts by 8.2%, 7.3%, 8.5%, and 1.0%, respectively. For GigaDataset, it averagely outperforms the counterparts by 5.8%, 7.1%, 4.4%, and 1.7%, respectively. These prove the strength of the proposed algorithm, which can be a robust and effective method for cross-subject motor imagery BCI.

The above is our research on the cross-subject problem in the same dataset. We will further our study to focus cross-dataset problems. That is to say, we want to select and transfer model trained with data from datasets to target subjects in another dataset. It will be more challenging and makes more sense for realistic applications. Imagine that, if data from any laboratory can be made full use of and utilized to train effective models for target users, the problem of being short of data and hard to train models for motor imagery BCI will vanish ultimately. It will provide more flexibility and robustness for BCI. We believe the BCI users will pay no effort for training and the BCI devices will be plug-and-play in the future.

## Figures and Tables

**Table 1:** Detailed architecture for the proposed DCJNN.

Layer	Input (ba × ch × *T*)^1^	Operations	Output
1	ch × *T*	32 × Conv1D (1 × 16)	32 × ch × *T*
	32 × ch × *T*	BatchNorm	32 × ch × *T*
	32 × ch × *T*	Dropout (0.2)	32 × ch × *T*

2	32 × ch × *T*	32 × Conv1D (ch × 1)	32 × 1 × *T*
	32 × 1 × *T*	BatchNorm	32 × 1 × *T*
	32 × 1 × *T*	Transpose	1 × 32 × *T*
	1 × 32 × *T*	Dropout (0.2)	1 × 32 × *T*

3	1 × 32 × *T*	16 × Conv2D (2 × 16)	16 × 32 × *T*
	16 × 32 × *T*	BatchNorm	16 × 32 × *T*
	16 × 32 × *T*	Maxpool2D (2 × 4)	16 × 16 × *T*/4

4	16 × 16 × *T*/4	4 × Conv2D (2 × 16)	4 × 16 × *T*/4
	4 × 16 × *T*/4	Maxpool2D (2 × 8)	4 × 8 × *T*/32

5	4 × 8 × *T*/32	Flatten	1 × (4 × 8 × *T*/32)

6	1 × (4 × 8 × *T*/32)	Softmax regression	(*N* × 1)^2^

^1^ba denotes the number of samples fed to the network each time. ch denotes the channel. *T* denotes the number of time points. ^2^*N* stands for the number of classes.

## Data Availability

The data used to support the findings of this study are included within the article. GrazA [46] can be found at URL: http://www.bbci.de/competition/iv/. The GigaDataset [47] in this paper can be found at URL: http://gigadb.org/dataset/100295.
